# Comparison of Signal‐ and Volume‐Based Ventilation‐Weighted Assessment Using 3D FLORET UTE MRI in Patients With Various Pulmonary Disease

**DOI:** 10.1002/mrm.70239

**Published:** 2026-02-03

**Authors:** Filip Klimeš, Joseph W. Plummer, Andreas Voskrebenzev, Marcel Gutberlet, Marius M. Klein, Matthew M. Willmering, Alexander M. Matheson, Abdullah S. Bdaiwi, Frank Wacker, Jason C. Woods, Zackary I. Cleveland, Laura L. Walkup, Jens Vogel‐Claussen

**Affiliations:** ^1^ Institute of Diagnostic and Interventional Radiology Hannover Medical School Hannover Germany; ^2^ Biomedical Research in Endstage and Obstructive Lung Disease Hannover (BREATH), German Center for Lung Research (DZL) Hannover Germany; ^3^ Department of Radiology, Charité ‐ Universitätsmedizin Berlin Freie Universität Berlin, Humboldt‐Universität zu Berlin, Berlin Institute of Health Berlin Germany; ^4^ Center for Pulmonary Imaging Research, Cincinnati Children's Hospital Medical Center Cincinnati Ohio USA; ^5^ Department of Biomedical Engineering University of Cincinnati Cincinnati Ohio USA; ^6^ Cardiovascular Branch, Division of Intramural Research National Heart, Lung, and Blood Institute, National Institutes of Health Bethesda Maryland USA; ^7^ Imaging Research Center, Department of Radiology Cincinnati Children's Hospital Medical Center Cincinnati Ohio USA; ^8^ Department of Pediatrics University of Cincinnati Medical Center Cincinnati Ohio USA; ^9^ Department of Physics University of Cincinnati Cincinnati Ohio USA

**Keywords:** FLORET, hyperpolarized ^129^Xe MRI, Jacobian, lung MRI, PREFUL, UTE, ventilation imaging

## Abstract

**Purpose:**

3D free‐breathing, proton, contrast‐agent‐free MR methods are increasingly used for pulmonary ventilation‐weighted measurements. The methods are split between: (1) signal‐based, which rely on lung parenchyma signal changes during respiration, and (2) volume‐based that utilize the Jacobian determinant of deformation fields from the image registration. This study compares both proton methods using respiratory‐resolved images acquired using fermat‐looped orthogonally encoded trajectories (FLORET) acquisition.

**Methods:**

Free‐breathing FLORET data were acquired from participants with various pulmonary conditions (*N* = 29) and healthy controls (*N* = 7), and reconstructed into respiratory phase‐resolved images. Signal‐based regional ventilation (RVent) was quantified using the 3D phase‐resolved functional lung algorithm, and volume‐based Jacobian ventilation (JVent) was derived as the Jacobian of the deformation field from the direct image registration of the end‐expiratory image to the end‐inspiratory image. Differences between the means, coefficients of variation (CoVs), and their ventilation defect percent (VDP) were quantified by Bland–Altman plots. The spatial overlap of the defect maps was determined by multi‐class Sørensen–Dice coefficient, and Spearman correlations to ^129^Xe MRI were assessed.

**Results:**

In all study participants, statistically significant differences were found between means/CoVs of RVent and JVent parameters (both *p* < 0.0001), but not VDP (*p* = 0.38). The median spatial overlap of the defect maps was 86%. VDP_RVent_ showed stronger correlation (*ρ* = 0.78, Meng *Z* = 4.36, *p* < 0.0001) to VDP_129Xe_ than JVent (*ρ* = 0.34).

**Conclusion:**

Although both proton lung MRI methods successfully identified ventilation defects, the stronger correlation between signal‐based and ^129^Xe MRI indicates that RVent may provide a more reliable assessment of lung ventilation in clinical applications in comparison to volume‐based parameters.

## 1 Introduction

Currently, chest computed tomography (CT) represents the state‐of‐the‐art modality to capture lung parenchyma pathology or monitor changes over time. Beyond morphological assessment, the use of CT has expanded further to evaluate lung function. Such advancements include quantification of air trapping and emphysema using parametric response mapping [1] or even pulmonary ventilation by spatially aligning inspiratory CT chest scan to the expiratory CT chest scan, and subsequent voxel‐wise analysis of the deformation field. In theory, voxel volume changes quantified by the Jacobian determinant (JD) of the deformation field depict the tissue expansion/shrinkage which corresponds directly to pulmonary ventilation [2]. It has been shown that regional ventilation quantified using JD has a strong correspondence with Galligas positron emission tomography ventilation measurements [3] and may serve as a potential biomarker for progression in patients with idiopathic pulmonary fibrosis [4].

On the other hand, free‐breathing 3D proton magnetic resonance imaging (MRI) techniques are also increasingly used for indirect ventilation‐weighted lung assessment, providing a radiation‐free alternative to chest CT at the cost of decreased spatial resolution and longer acquisition times. These methods include: (1) signal‐based methods, which track lung signal intensity changes to compute regional ventilation (RVent) [5, 6, 7]; and (2) volume‐based methods, which borrow concepts from CT and use the JD of the deformation field to measure ventilation (JVent) [8, 9]. A recent study showed that RVent, derived using 2D proton imaging, correlates stronger with CT‐based parametric response mapping, suggesting it offers a more reliable assessment of lung ventilation [10]. Given the complexity of 3D lung respiratory motion and advancements in ultrashort echo times (UTE) techniques, that generally provide higher signal‐to‐noise characteristics in lung parenchyma, 3D image registration should theoretically yield improved results with the JVent method. Recent studies have demonstrated a correspondence between signal‐based RVent methods with ^129^Xe MRI in patients with chronic obstructive lung disease [11] and children with cystic fibrosis [12]. In addition, agreement of volume‐based JVent methods with ^129^Xe MRI has been shown in both pediatric and adult cystic fibrosis populations [13]. However, the correspondence between the two proton‐based methods remains unknown.

Therefore, the aim of this study is to compare the performance of RVent and JVent ventilation surrogates against the direct measurement of ventilation using ^129^Xe MRI and current clinical standard of pulmonary function tests (PFTs) in people with distinct pulmonary disease and healthy volunteers.

## 2 Methods

### 2.1 Study Participants

All studies were registered under US Food and Drug Administration (IND‐123577), and were approved by the Cincinnati Children's Institutional Review Board. Written informed consent was obtained from all adult participants or parents of pediatric subjects, and age‐appropriate assent was obtained from pediatric participants only. A total of 29 participants with pulmonary diseases (16 male, 13 female, age range: 7–55), including those with cystic fibrosis (CF, *N* = 14, age range: 7–22, 8 male, 6 female), lymphangioleiomyomatosis (LAM, *N* = 7, age range: 39–55, 7 female), post hematopoietic stem cell transplant (post‐HSCT, *N* = 4, age range: 11–21, 4 male), bronchiolitis obliterans syndrome (BOS, *N* = 3, age range: 14–21, 3 male), and neuroendocrine cell hyperplasia of infancy (NEHI, *N* = 1, age: 13, male), and 7 healthy participants (age range: 8–27, 5 male, 2 female) were included in this study. Inclusion criteria were age greater than 7 years and the ability to comply with a breath‐hold. Exclusion criteria included an active respiratory infection, chest tightness, or sinus infection within 1 week prior to MRI; baseline pulse oximetry (SpO_2_) ≤ 95%; pregnancy or a positive urine pregnancy test in females of reproductive age; and standard contraindications to MRI [14].

### 2.2 Image Acquisition and Postprocessing

The free‐breathing proton and breath‐hold ^129^Xe imaging was performed on a Philips Ingenia 3T system (Philips Medical Systems, Best, The Netherlands).

#### 2.2.1 Free Breathing Proton MRI

Proton UTE MRI data were acquired using a 3D Fermat looped, orthogonally encoded trajectories (FLORET) sequence, as described by Willmering et al. [15] Acquisition parameters include: TE/TR 0.1/3.76 ms, readout length = 0.95 ms, flip angle = 4.0°, 140 000 ± 20 000 excitations, FOV of 320 ± 40 mm^3^, and acquisition resolution = (1.6 mm)^3^, with *k*‐space continuously acquired for 7 ± 2 min using a 16‐channel anterior coil and a 12‐channel posterior coil. As described in a previous publication [14], *k*‐space data were binned into 24 respiratory states using the respiratory bellows signal and the final respiratory‐resolved data were reconstructed to a spatial resolution of 3 mm^3^ [9]. This step was followed by the image registration of the images from the whole respiratory cycle to the end‐inspiratory image using advanced normalization tools (ANTs) [16]. During image registration, an initial affine transformation was followed by nonrigid, flexible registration. The nonrigid step used a B‐spline symmetric normalization diffeomorphic algorithm with the following parameters: spline order = 3, metric = cross‐correlation with a radius of 4 and metric weight of 1, gradient step = 1, convergence criteria = 150 × 150 × 100 × 80 iterations with a tolerance of 1 × 10^−6^ over 10 iterations, shrink factors = 6 × 4 × 2 × 1, smoothing sigmas = 3 × 2 × 1 × 0, updated field mesh size at base level = 40, and total field mesh size at base level = 0. A rectangular mask encompassing the full 3D lung volume was applied for both rigid and nonrigid transformations to reduce convergence time. All ANTs commands were executed via Matlab (The MathWorks Inc., Release R2021a, Natick, MA, USA). Ventilation maps were derived from:

1. Signal‐based regional ventilation (RVent) [17]

$$\text{RVent}=S_{\mathrm{Reg}}*\frac{\left(S_{\mathrm{Exp}}-S_{\text{Insp}}\right)}{\left(S_{\text{Insp}}*S_{\mathrm{Exp}}\right)},$$

with target registration (Reg), expiration (Exp), and inspiration (Insp) signals (S). Since the target image for registration was the same as the inspiration image, the previous formula can be simplified to:

$$\text{RVent}=\frac{\left(S_{\mathrm{Exp}}-S_{\text{Insp}}\right)}{S_{\mathrm{Exp}}}.$$

This formula is commonly referred to as fractional ventilation [18]. For the RVent calculation, image filtering and denoising were performed following a previously published approach [14].

2. Deformation field obtained through direct image registration of the end‐expiratory image to the target end‐inspiratory image, producing volume‐based ventilation, JVent, calculated as follows:

JVent=det(J)−1,

With det(*J*) representing the JD [19].

A median filter of matrix size (7 × 7 × 7) voxels was applied in the spatial domain to increase the signal‐to‐noise ratio of JVent maps.

#### 2.2.2 ^129^Xe MRI

^129^Xe acquisitions were carried out with a vest coil (Clinical MR Solutions, Brookfield, WI, USA), using enriched (> 85%) ^129^Xe gas polarized to 20%–40% using a Polarean 9820A ^129^Xe polarizer (Polarean Imaging PLC, Durham, NC, USA). The dose of ^129^Xe was set to 1/6 of the subjects' sex‐ and height‐predicted total lung capacity (not exceeding 1 L) for people with CF [20], and 1/6 of the predicted forced vital capacity (not exceeding 1 L) for the rest of study participants [21]. Doses were stored in Tedlar bags (Jensen Inert, Coral Springs, FL, USA) and administered by inhaling from functional residual capacity (FRC).

^129^Xe images were acquired with a multi‐slice 2D spoiled gradient‐echo sequence: TE/TR < 5/< 10 ms, FOV_*xy*_ (300–400 mm)^2^, FOV_z_ (150–200 mm), spatial resolution (3 × 3 × 15 mm)^3^, slice gap 0 mm, and scan duration < 15 s breath‐hold. Prior to the ^129^Xe ventilation acquisition, calibration scans were performed to determine the optimal flip angle (8°–20°) for each subject's ^129^Xe ventilation acquisition, with modifications based on each individual's number of excitations and receiver frequency.

For the VDP calculations, the ^129^Xe images were segmented using a pretrained deep learning network [22] and corrected for B_1_ inhomogeneity using N4 bias field correction [23]. VDP_129Xe_ was calculated from an established threshold as the percentage of the lung voxels with signal intensity below 60% of the mean whole‐lung signal intensity [24, 25].

### 2.3 Pulmonary Function Tests

Pulmonary function tests (PFTs) were performed according to current American Thoracic Society/European Respiratory Society guidelines [26]. The assessed parameters included percent predicted forced expiratory volume in 1 s (ppFEV_1_), percent predicted forced vital capacity (ppFVC), and the FEV_1_/FVC ratio.

### 2.4 Statistical and Image Analysis

Image and statistical analysis were performed in Matlab (The MathWorks Inc., Release R2022b, Natick, MA, USA).

The distributions of all MRI‐derived parameters were tested for normality using the Lilliefors test. As most of the parameters showed non‐normal distribution, non‐parametric tests were used. Unless otherwise stated, the evaluated data are presented as median with interquartile ranges.

For proton MRI, lung parenchyma regions were segmented at end‐inspiration prior to analysis using a trained convolutional neural network, and verified by expert readers (JWP, 7 years; and FK, 9 years of experience in pulmonary MRI).

Mean and coefficient of variation (CoV) for RVent/JVent across the whole lung were calculated per subject within the lung parenchyma mask. Subsequently, for both RVent/JVent maps, threshold analysis was performed on all included study participants to find the optimal threshold to distinguish between healthy and diseased participants. For each defined threshold, VDP values were calculated whereby all RVent/JVent values below that specific threshold were considered as ventilation defects. Following that, the receiver operating characteristic (ROC) analysis on those VDP values was performed to determine the area under the curve (AUC) and the optimal operating point of the ROC curve. The operating point was used to re‐label the subjects into “healthy” and “disease” and these labels were compared to the ground truth labels. This led to computation of sensitivity, specificity, and Youden's Index. Additionally, the Spearman correlation evaluated the strength of the relationship to VDP_129Xe_. For each RVent/JVent parameter map, two threshold methods were considered:

1. A fixed threshold defined as a ventilation‐weighted value ranging from 0.01 to 0.15, incremented by a 0.01 step size,
2. A flexible threshold defined as the 90th percentile of all ventilation‐weighted values within the lung parenchyma, multiplied by a factor ranging from 0.1 to 0.9. The multiplication factor increased with a step size of 0.01 between 0.1 and 0.2, and with a step size of 0.05 between 0.2 and 0.9.

Selection criteria for the optimal threshold for RVent/JVent maps were high sensitivity, specificity, Youden's Index, AUC, and strong correlation to VDP from ^129^Xe MRI (VDP_129Xe_). Also, the aim was to have median VDP values < 7% for healthy participants and a pronounced VDP difference between healthy and diseased participants.

The VDP values determined using the optimal thresholds along with mean and CoV of RVent/JVent were compared using paired Wilcoxon‐signed rank tests and Bland–Altman plots. Further, relationships between RVent and JVent parameters were assessed by Spearman correlation. Spatial overlap between both proton defect maps was assessed by the spatial overlap metric and Sørensen‐Dice coefficient for healthy and disease areas. The spatial overlap was defined as the percentage of matching voxels labeled as either ventilated (healthy) or non‐ventilated (defect) between the proton defect maps

$$\text{Overlap}=\left(\frac{2*\left(n_{\mathrm{vv}}+n_{\mathrm{dd}}\right)}{n_{\text{vRVent}}+n_{\text{vJvent}}+n_{\text{dRVent}}+n_{\text{dJVent}}}\right)*100,$$

where *n*_vv_ represents number of voxels labeled as ventilated in both maps, and *n*_dd_ number of voxels labeled as defect in both maps. *n*_vRVent_ and *n*_vJVent_ denote the number of ventilated voxels in RVent‐based defect maps and JVent‐based defect maps, respectively; *n*_dRVent_, *n*_dJVent_ denote the number of defect voxels in RVent‐based and JVent‐based defect maps, respectively.

To assess the relationship to the direct ventilation measurement, Spearman correlations were calculated between proton VDPs and VDP_129Xe_ and the bias was quantified by Bland Altman plots and tested for significance using the paired Wilcoxon signed rank test. To compare between the Spearman correlation coefficients, a Meng's *z*‐test was performed [27].

Additionally, the SNR for RVent and JVent maps was computed on the central coronal slice at the level of the tracheal bifurcation, using the morphological image that served as the fixed reference for registration. Regions of interest were placed within the lung parenchyma mask and in the background to measure signal and noise, respectively. The differences between the SNR values were quantified by Bland–Altman analysis and tested for significance using the paired Wilcoxon signed rank test.

Correspondence of all MRI‐derived parameters to measures from PFT was assessed by Spearman correlation analysis.

To determine the discriminative power of each method, AUC differences between all methods were tested for significance using the DeLong method [28].

For all comparisons and statistical tests, *p* values < 0.05 were deemed significant.

## 3 Results

### 3.1 Threshold Analysis for Proton MRI

For RVent, a flexible threshold defined as the 90th percentile multiplied by a factor of 0.25 was selected, yielding a sensitivity of 0.93, specificity of 0.86, a Youden index of 0.79, and a maximum AUC of 0.92. For JVent, a flexible threshold of the 90th percentile multiplied by a factor of 0.16 was chosen, resulting in a sensitivity of 0.66, specificity of 1.00, a Youden index of 0.66, and an AUC of 0.78. Complete results of the threshold analysis are listed in Table S1 for RVent and Table S2 for JVent parameters.

### 3.2 Comparison Between RVent and JVent Maps

**FIGURE 1:**
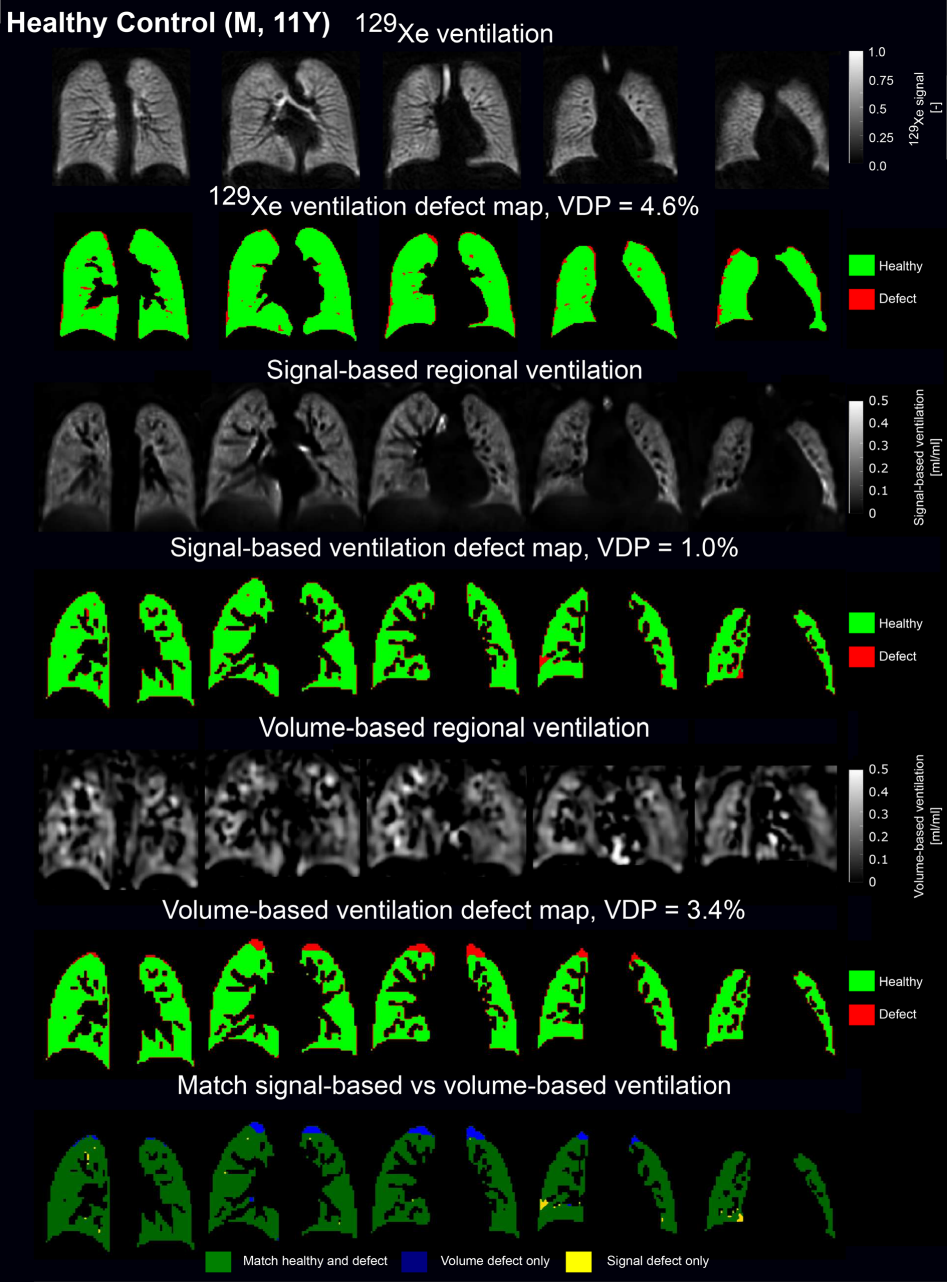
^129^Xe ventilation and ventilation defect images (1st and 2nd rows) and corresponding proton ventilation parameter maps of a healthy subject (11‐year‐old male, ppFEV_1_ = 100.5). The 3rd and 4th rows display the signal‐based RVent map alongside its defect maps, while the 5th and 6th rows present the unfiltered volume‐based JVent map with its corresponding defect map. The 7th row presents spatial agreement between ventilation defect maps of both proton techniques, with a total spatial overlap of 95.6% across all evaluated slices. Matched defect and healthy areas are shown in dark green, with differences highlighted in blue and yellow.

**FIGURE 2:**
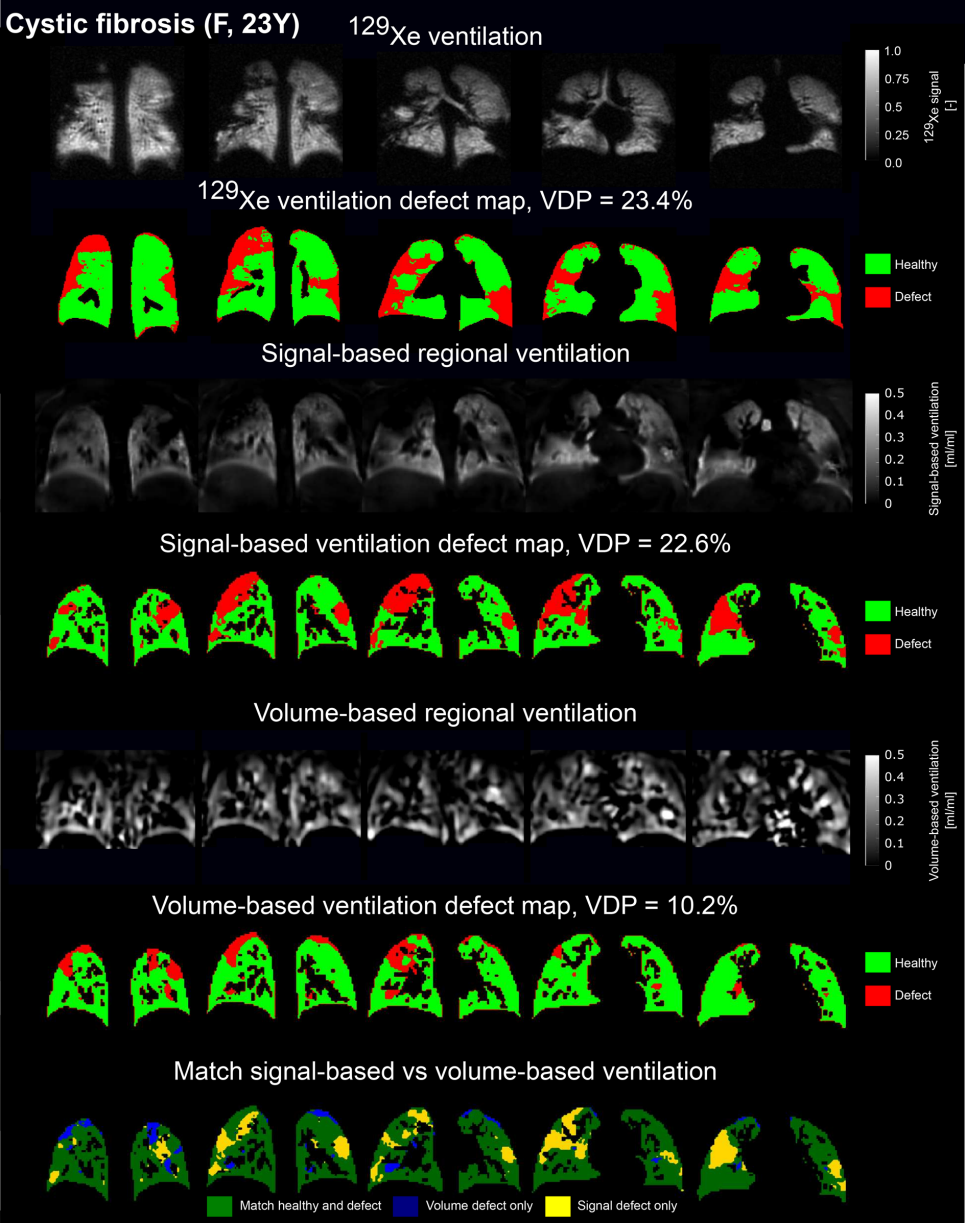
^129^Xe ventilation and ventilation defect images (1st and 2nd rows) and corresponding proton ventilation maps of a cystic fibrosis participant (23‐year‐old female, ppFEV_1_ = 89.8). The 3rd and 4th rows display the signal‐based RVent map alongside its defect maps, while the 5th and 6th rows present the unfiltered volume‐based JVent map with its corresponding defect map. The 7th row presents spatial agreement between ventilation defect maps of both proton techniques, with a total spatial overlap of 79.0% across all evaluated slices. Matched defect and healthy areas are shown in dark green, with differences highlighted in blue and yellow.

Figures 1 and 2 show representative proton ventilation parameter maps for a healthy and a cystic fibrosis participant, alongside ^129^Xe images. The RVent maps visually correlate more closely with ^129^Xe imaging than JVent.

**FIGURE 3:**
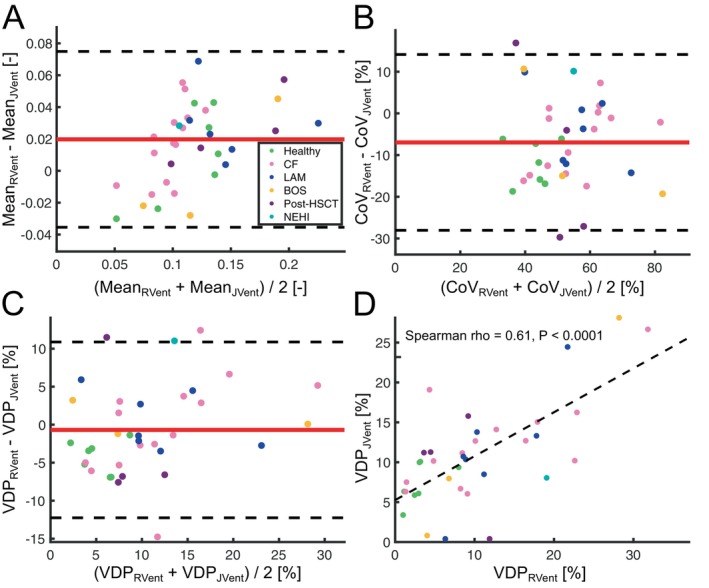
Bland–Altman analysis of proton methods along with regression analysis of their VDP values. In the Bland–Altman plots, the red line represents the mean bias, while the dotted black lines indicate the limits of agreement, calculated as the mean bias±1.96 standard deviations. In the regression plots, the black dotted line represents the fitted regression line. In (A), Mean_RVent_ versus Mean_JVent_; in (B), CoV_RVent_ versus CoV_JVent_; in (C), VDP_RVent_ versus VDP_JVent_; in (D), regression analysis between VDP_RVent_ and VDP_JVent_.

Statistically significant differences were found between mean and CoV values of RVent and JVent across all study participants and diseased participants only (mean bias of 0.02 mL/mL for the mean and −6.96% for CoV, respectively; all *p* < 0.0001; see Figure 3a,b and Table 1). However, no significant difference was observed in VDP values between RVent and JVent for both comparisons (both *p* ≥ 0.38, Figure 3c, Table 1). Notably, there was a negative bias of 4.20% for VDP_JVent_ and 11.8% for CoV JVent when compared to corresponding RVent parameters in the healthy participant group (both *p* = 0.0156, Table 1). A positive bias was observed in the SNR of JVent relative to RVent maps across all participants (mean bias of 2.65, *p* = 0.0031), as well as within the subgroup of diseased participants (mean bias of 3.81, *p* = 0.0015).

**TABLE 1:** Analysis of proton MRI lung ventilation quantification methods.

		Descriptive statistics		Bland–Altman analysis		Spearman correlation	
	Parameter	RVent	JVent	Absolute mean difference (LoA)	*p* ^a^	Spearman rho	*p* ^b^
All participants (*N* = 36)	Mean (mL/mL)	0.13 (0.10–0.15)	0.10 (0.09–0.12)	0.02 (−0.04–0.07)	< 0.0001*	0.65	< 0.0001*
	CoV (%)	46.6 (40.4–59.9)	57.2 (48.6–62.4)	−6.96 (−28.05–14.12)	< 0.0001*	0.61	< 0.0001*
	VDP (%)	8.5 (3.9–13.7)	10.2 (6.6–13.4)	−0.69 (−12.26–10.88)	0.38	0.61	< 0.0001*
	SNR (−)	1.9 (1.3–2.8)	4.4 (2.5–7.7)	2.65 (−27.21–32.50)	0.0031*	—	—
Healthy participants (*N* = 7)	Mean (mL/mL)	0.14 (0.11–0.14)	0.11 (0.10–0.13)	0.01 (−0.05–0.07)	0.47	0.43	0.35
	CoV (%)	37.7 (33.3–39.0)	50.1 (46.2–53.4)	−11.82 (−22.39–1.24)	0.0156*	0.54	0.24
	VDP (%)	3.0 (1.8–3.1)	6.3 (6.0–9.7)	−4.20 (−8.48–0.09)	0.0156*	0.79	0.0480*
	SNR (−)	2.9 (2.6–3.5)	3.5 (1.2–5.7)	−2.16 (−29.37–25.04)	0.94	—	—
Non‐healthy participants (*N* = 29)	Mean (mL/mL)	0.13 (0.10–0.15)	0.10 (0.09–0.12)	0.02 (−0.03–0.06)	< 0.0001*	0.61	< 0.0001*
	CoV (%)	48.5 (44.9–62.6)	59.0 (49.9–63.2)	−5.79 (−28.24–16.65)	0.0141*	0.54	0.0029*
	VDP (%)	9.2 (6.3–17.8)	11.2 (8.0–14.1)	0.16 (−12.03–12.35)	0.89	0.54	0.0029*
	SNR (−)	1.8 (1.3–2.2)	4.5 (2.6–8.2)	3.81 (−26.65–34.27)	0.0015*	—	—

*Note*: Ventilation parameter values are expressed as a median with interquartile ranges. Statistically significant results between paired comparisons between signal‐based (RVent) and volume‐based (JVent) parameters are marked with *.

Abbreviations: CoV, coefficient of variation; JVent, volume‐based ventilation based on Jacobian determinant; LoA, levels of agreement; RVent, signal‐based regional ventilation; VDP, ventilation defect percentage.

^a^ *p* value determined using paired Wilcoxon signed rank test.

^b^ *p* value determined using Fisher's *z*‐transformation.

In all study participants, all RVent parameters were significantly correlated to their corresponding JVent parameters (all *ρ* ≥ 0.61, all *p* < 0.0001, Table 1).

The proton defect maps showed a median spatial overlap of 85.7% for all study participants with generally lower values for diseased participants (median spatial overlap of 91.0% for healthy vs. 83.3% for diseased participants, respectively; Table 2). Median Sørensen–Dice coefficients for defects were 0.13 in the full cohort, 0.05 in healthy participants only, and 0.17 in people with pulmonary disease. For healthy areas, the median Sørensen–Dice coefficient was 0.92 for all study participants, 0.95 for healthy participants only, and 0.91 for people with pulmonary disease.

**TABLE 2:** Spatial overlap analysis between proton defect maps derived from signal‐based RVent and volume‐based JVent maps.

	Spatial overlap (%)	Sørensen–Dicedisease (−)	Sørensen–Dicehealthy (−)
All participants	85.8 (75.9–88.4)	0.13 (0.07–0.28)	0.92 (0.88–0.94)
Healthy participants	91.0 (87.7–92.6)	0.05 (0.02–0.08)	0.95 (0.93–0.96)
Non‐healthy participants	83.3 (79.0–87.5)	0.17 (0.10–0.31)	0.91 (0.87–0.93)

*Note*: The spatial overlap of the defect maps was determined by multi‐class Sørensen–Dice coefficient.

### 3.3 Relationship to ^129^Xe MRI

**FIGURE 4:**
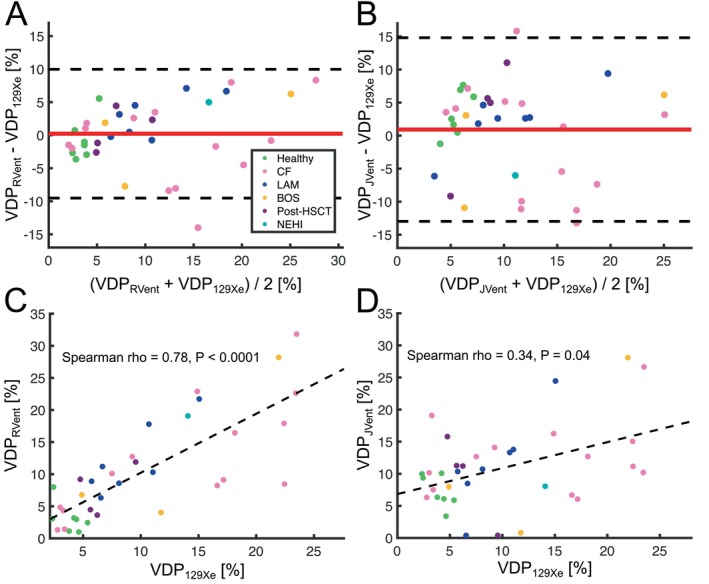
Bland–Altman analysis between proton methods and ^129^Xe MRI along with regression analysis of their VDP values. In the Bland–Altman plots, the red line represents the mean bias, while the dotted black lines indicate the limits of agreement, calculated as the mean bias±1.96 standard deviations. In the regression plots, the black dotted line represents the fitted regression line. In (A), Bland–Altman plot VDP_RVent_ versus VDP_129Xe_; in (B), Bland–Altman plot VDP_JVent_ versus VDP_129Xe_; in (C), regression analysis VDP_RVent_ versus VDP_129Xe_; in (D), regression analysis between VDP_JVent_ and VDP_129Xe_.

Significant correlations were found between CoV_RVent_, VDP_RVent_, CoV_JVent_, VDP_JVent_, and VDP_129Xe_ (all *ρ* > 0.34, all *p* < 0.0403, Table 3). There was no significant bias between either VDP_RVent_ or VDP_JVent_ to VDP_129Xe_ (both *p* ≥ 0.41, Figure 4a,b, Table 3). The levels of agreement (LoA) were broader in the comparison between VDP_JVent_ and VDP_129Xe_ when compared to the comparison between VDP_RVent_ and VDP_129Xe_. Figure 4c,d shows the regression analyses of VDP_RVent_ versus VDP_129Xe_, and VDP_JVent_ versus VDP_129Xe_, respectively. VDP_RVent_ was more strongly correlated with VDP_129Xe_ (*ρ* = 0.78) than VDP_JVent_ was with VDP_129Xe_ (*ρ* = 0.34), as confirmed by Meng's *Z* test (*Z* = 4.36, *p* < 0.0001).

**TABLE 3:** Spearman correlation analysis of RVent/JVent ventilation parameters to gold standard ventilation measurement of ^129^Xe MRI and its ventilation defect percentage values.

Parameter (*N* = 36)	VDP_129Xe_			
	Spearmanrho	*p* ^b^	Mean bias (LoA)	*p* ^a^
Mean_RVent_	−0.07	0.70	—	—
CoV_RVent_	0.76	< 0.0001*	—	—
VDP_RVent_	0.78	< 0.0001*	0.23 (−9.52–9.97)	0.64
Mean_JVent_	−0.13	0.43	—	—
CoV_JVent_	0.34	0.0437*	—	—
VDP_JVent_	0.34	0.0403*	0.92 (−12.98–14.81)	0.41

*Note*: Statistically significant correlations are marked with *. Additionally, the mean bias was quantified using Bland–Altman plots and was calculated as the difference between VDP_RVent_ (or VDP_JVent_) and VDP_129Xe_.

Abbreviations: CoV, coefficient of variation; JVent, volume‐based ventilation based on Jacobian determinant; LoA, levels of agreement; RVent, signal‐based regional ventilation; VDP, ventilation defect percentage.

^a^ *p* determined using paired Wilcoxon signed rank test.

^b^ *p* determined using Fisher's z‐transformation.

### 3.4 Relationship of MRI‐Based Parameters to PFT

PFT data were not recorded for three healthy volunteers, one LAM subject, and one NEHI patient. Across all participants with PFT (*N* = 31), the median ppFEV_1_ was 92.00 with interquartile range (IQR) between 82.77%–107.40%, the median ppFVC was 100.70 (IQR: 90.18–108.65)%, and the median FEV1/FVC ratio was 82.00 (IQR: 74.85–88.59). In the diseased cohort with PFT (*N* = 27), the median ppFEV1 was 89.80 (IQR: 81.85–106.10)%, ppFVC was 99.82 (IQR: 88.15–108.65)%, and the FEV1/FVC ratio was 81.57 (IQR: 74.85–86.09). Among healthy controls with PFT (*N* = 4), the median ppFEV1 was 104.85 (IQR: 99.35–110.40)%, ppFVC was 104.00 (IQR: 102.03–106.88)%, and the FEV1/FVC ratio was 88.23 (IQR: 81.27–94.07).

There were significant correlations between CoV_RVent_, VDP_RVent_, and VDP_129Xe_ with ppFEV_1_ and FEV_1_/FVC (all *ρ* ≤ −0.44, all *p* ≤ 0.0131). A significant correlation was also observed between mean RVent and ppFVC (*ρ* = −0.43, *p* = 0.0153). No significant correlations were found between all Jacobian‐derived parameters and all PFT measurements (all *p* ≥ 0.06). Complete results are presented in Table 4.

**TABLE 4:** Spearman correlation analysis of RVent/JVent and ^129^Xe MRI‐derived ventilation parameters to pulmonary function test parameters (*N* = 31, 27 non‐healthy, 4 healthy).

Parameter (*N* = 31)	ppFEV_1_		ppFVC		FEV_1_/FVC	
	Spearmanrho	*p* ^a^	Spearman rho	*p* ^a^	Spearman rho	*p* ^a^
VDP_129Xe_	−0.65	< 0.0001*	−0.26	0.16	−0.56	0.0010*
Mean_RVent_	−0.30	0.11	−0.43	0.0153*	−0.11	0.54
CoV_RVent_	−0.47	0.0081*	−0.12	0.53	−0.48	0.0063*
VDP_RVent_	−0.47	0.0073*	−0.16	0.40	−0.44	0.0131*
Mean_JVent_	−0.18	0.32	−0.20	0.27	−0.11	0.56
CoV_JVent_	−0.19	0.31	0.05	0.80	−0.33	0.07
VDP_JVent_	−0.26	0.15	−0.03	0.86	−0.34	0.06

*Note*: Statistically significant correlations are marked with *.

Abbreviations: CoV, coefficient of variation; JVent, volume‐based ventilation based on Jacobian determinant; ppFEV_1_, percent predicted forced expiratory volume in 1 s; ppFVC, percent predicted forced vital capacity; RVent, signal‐based regional ventilation; VDP, ventilation defect percentage.

^a^ *p* value determined using Fisher's *z*‐transformation.

### 3.5 Discriminative Power

**FIGURE 5:**
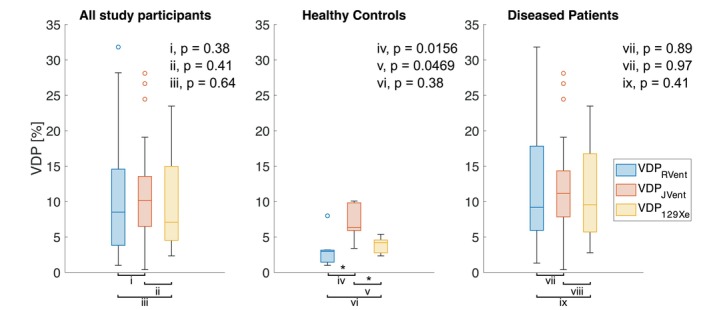
Boxplots showing the VDP values distribution in all study participants (left), in healthy participants only (middle), and in diseased participants only (right). Note significantly higher VDP values in the healthy control group for VDP_JVent_ when compared to VDP_RVent_ and VDP_129Xe_.

VDP distributions across the whole cohort and healthy/disease groups are shown in Figure 5. Significantly higher VDP_JVent_ values were found in the healthy control group when compared to VDP_RVent_ and VDP_129Xe_ (both *p* ≤ 0.0469).

**FIGURE 6:**
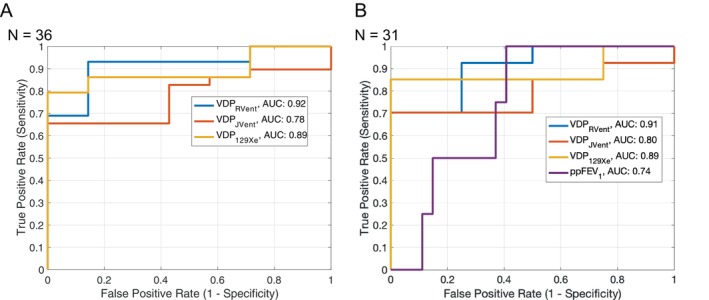
ROC analysis displays the three VDP parameters and pulmonary function test derived ppFEV_1_ along with their respective AUC values, highlighting the superior performance of VDP_RVent_ compared to VDP_JVent_. In (a) ROC analysis for all study participants (*N* = 36) and in (b) ROC analysis in study participants where pulmonary function tests were available (*N* = 31). Note the better differentiation between healthy and diseased patients with VDP_RVent_ and VDP_129Xe_ compared to VDP_JVent_ in (a) and (b) and ppFEV_1_ in (b).

In all study participants (*N* = 36), AUC value was lower for VDP_JVent_ (0.78) compared to VDP_129Xe_ (0.89) and VDP_RVent_ (0.92, Figure 6a,b). However, the DeLong method revealed no significant differences in the performance of all three methods (all *p* ≥ 0.06).

In the subset of participants who underwent both spirometry and MRI combined (*N* = 31), no significant differences were observed (all *p* ≥ 0.09). The AUC value for ppFEV_1_ (0.74) was the lowest (Figure 6b), compared to the AUC values of MRI‐derived parameters: 0.80 for VDP_JVent_, 0.89 for VDP_129Xe_ and 0.91 for VDP_RVent_.

## 4 Discussion

Understanding regional lung ventilation is critical to detect and monitor pulmonary abnormalities. Among respiratory‐resolved proton UTE MRI approaches, both Jacobian‐based ventilation (JVent) derived from image registration and signal‐based ventilation (RVent) derived from dynamic signal changes offer complementary pathways to estimate ventilation. In theory, RVent and JVent should yield similar results, as both quantify regional lung volume changes during respiration. While JVent captures geometric expansion derived from image registration, RVent reflects the corresponding change in air content. Ideally, these measures should align closely; however, discrepancies can arise due to physiological differences and technical factors such as registration accuracy, signal‐to‐noise ratio, and susceptibility effects. Despite their shared goal, differences in their underlying assumptions, sensitivity to noise, and image processing demands can lead to varying results and require careful interpretation. Therefore, a direct comparison of these methods is essential to assess their relative accuracy, reliability, and clinical utility, particularly in relation to established benchmarks like hyperpolarized ^129^Xe MRI. The goal of this work is to comprehensively compare these approaches against hyperpolarized ^129^Xe MRI.

The results indicate significant differences between the RVent and JVent methods for assessing lung ventilation via 3D proton FLORET UTE MRI. VDP_RVent_ demonstrated a stronger correlation with ^129^Xe MRI and PFTs, likely due to inferior image quality of the JVent maps. Volume‐based JVent ventilation displayed a blurrier appearance, as previously reported [29], and showed a weaker correlation with ^129^Xe MRI. In the development of the JVent VDP calculation in this study, it was necessary to additionally apply a median filter of 7 × 7 × 7 to derive meaningful VDP values in the expected range while also maintaining correlation to ^129^Xe MRI. We also performed threshold analysis on unfiltered JVent maps as well as those processed with 3 × 3 × 3 and 5 × 5 × 5 median filters. However, the 7 × 7 × 7 filter yielded superior AUC values and produced VDP measurements within the expected healthy range for the control group in our study. This filter may have contributed to the observed blurring as well as higher SNR of the JVent maps.

Threshold analysis was performed to derive the most suitable thresholds for RVent/JVent parameters. Since a variety of pulmonary conditions were included, the determined threshold should be applicable for different pulmonary diseases. For VDP_RVent_, the determined threshold of the 90th percentile multiplied by a factor of 0.25 differs from the previously published threshold (90th percentile multiplied by a factor of 0.4) for 3D phase‐resolved functional lung (PREFUL) MRI [6], which showed responsiveness to therapy in CF [30] as well as spatial correspondence to direct ventilation imaging with dynamic ^19^F MRI [31] and breath‐hold ^129^Xe MRI [11]. A threshold considered in this work theoretically means to be less sensitive to ventilation inhomogeneities/changes but also less susceptible to noise. On the other hand, the correlation with ^129^Xe MRI was maximized, and the VDP values of healthy participants were in median below 3%, in contrast to earlier publications (reporting 6%–7% VDP for healthy) with the previously established threshold [32]. This also enabled better VDP matching closer to the ^129^Xe MRI VDP values reported for healthy volunteers (mean VDP of 1.6% ± 1.2%) in a previous study [33]. For the VDP_JVent_ values, we used a binary threshold in contrast to a previous study, which used the k‐means clustering methods [13]. While the authors report systematically higher VDP_JVent_ by 5% when compared to VDP_129Xe_ in people with CF, we observed no bias (mean difference of 0.9%) using the optimized threshold. Our observations are in line with a recent publication, which showed significant differences between binary and *k*‐means thresholding techniques for ^129^Xe MRI, with *k*‐means methods consistently showing systematically higher VDP values in 175 study participants consisting of healthy subjects, asthma and chronic obstructive pulmonary disease patients [34].

Both proton methods had similar VDP values, and their corresponding values showed a close relationship as highlighted by a strong positive correlation between them. This result was expected, as both proton methods are performed using the same images and their thresholds were adjusted to produce values within the anticipated range. While the volume‐based methods rely solely on the precise vector estimation during image registration (even small errors in vector calculation can lead to inaccurate results), signal‐based methods require mainly accurate bulk registration (i.e., groups of similar voxels being mapped onto similar voxels) and can further benefit from additional postprocessing parts, such as low‐pass filtering and image guided filtering, which may further improve the signal related to ventilation. Also, the signal‐based methods (such as PREFUL [35] MRI or 3D flow capacity‐weighted UTE MRI [7]) enable evaluation of the whole respiratory cycle, further adding value to these methods, as those dynamic metrics have been shown to be sensitive for prediction of future lung transplant loss [36] or changes associated with post‐COVID‐19 condition [37]. Analysis of the whole respiratory cycle using the volume‐based methods has been performed in healthy controls, employing parameters such as tidal volume and spontaneous peak expiratory flow [8, 38], but has not yet been applied to any patient population. However, the dynamic parameters derived from the signal‐based approach might also be of interest for volume‐based approaches and vice versa, both for integration into their analysis and for direct comparison between the two approaches. In this work, the flow‐volume loop analysis of the whole respiratory cycle was not integrated as we primarily concentrated on static ventilation imaging, which is more closely related to the breath‐hold imaging performed using ^129^Xe MRI.

The total spatial agreement between both proton methods was strong (median of 86%); however, the Sørensen–Dice coefficient in the defect areas of the diseased study participants was low (median value of 0.17). This number is likely biased by the class imbalance as there were low VDP values of both ventilation‐weighted measurements (both medians < 11.2%).

In this study, both proton ventilation‐weighted approaches were significantly correlated to ^129^Xe MRI, which represented direct measurement of ventilation. A similar strength of correlation in the range 0.61–0.82 was recently observed between 3D proton methods derived VDP_RVent_ and VDP_129Xe_ [12, 14]. As for the volume‐based method, a previous study [13] reported a higher correlation value of 0.64 between VDP_JVent_ and VDP_129Xe_ in contrast to our results. While the study used the same registration toolbox, there are differences in the fixed image chosen for registration, as the authors used registration towards the end‐expiratory state and in our work, image registration towards the end‐inspiratory image was performed. The choice of choosing the end‐inspiratory image from the tidal breathing as a fixed image for the image registration was motivated by the similar breathing position to the breath‐hold image (FRC + up to 1L of ^129^Xe gas mixture dose) acquired for ^129^Xe imaging. Additionally, the authors reported Pearson correlation, which is in contrast to the Spearman correlation used for our non‐normally distributed data. Apart from those technical differences, the study populations were different, as the previous study mainly assessed people with cystic fibrosis and for our study, we had a broader spectrum of pulmonary diseases and ages. Future research could examine patients with other pulmonary conditions and explore the performance of different registration algorithms to improve the image quality of JVent maps.

JVent VDP values of healthy volunteers were significantly higher than those derived from RVent and ^129^Xe MRI. In the previous publication, authors observed a similar trend between VDP_JVent_ and VDP_129Xe_ MRI in a group consisting of 12 healthy volunteers [13]. This finding, along with the superior AUC values of RVent, underscores RVent's potential to better distinguish between healthy and disease areas. This finding was supported by correlations to PFT data, where RVent showed significant correlations to ppFEV_1_ and FEV_1_/FVC, while JVent did not. A recent publication reported a significant correlation (Pearson *r* = −0.54) between the JVent parameter and ppFEV1. However, that study included 12 healthy volunteers and 45 participants with cystic fibrosis only [13]. While both RVent and JVent are sensitive to ventilation changes in obstructive and restrictive lung diseases, we assume that the JVent method is more sensitive to detecting larger structural or functional abnormalities, such as those commonly observed in cystic fibrosis, and therefore may be better suited for assessing restrictive disease patterns. In contrast, our study also included patients with more subtle, focal defects, which may be more challenging for JVent to capture accurately due to blurring artifacts present in the JVent maps. This may explain the lower, statistically non‐significant correlations between JVent and PFT measurements observed in our study. Consequently, the signal‐based RVent parameter may be more suitable for obstructive disease, where a more heterogeneous or patchy ventilation pattern is expected.

Means, CoVs, and VDPs of RVent/JVent capture complementary aspects of regional lung function. Biomarkers reflecting ventilation heterogeneity, such as CoV and VDP, potentially tend to correlate more strongly with FEV_1_, as both are influenced by airway obstruction. In contrast, metrics reflecting global volume change, like mean RVent and mean JVent, are more closely related to FVC, which represents total ventilated lung volume rather than airflow dynamics. The choice of biomarker for clinical practice may therefore depend on the clinical question. In previous studies [12, 30], CoV and VDP values of RVent have demonstrated higher sensitivity for disease detection and monitoring, while mean RVent/JVent values may be influenced by respiratory rate and tidal volume [39], limiting their sensitivity.

While other sequences such as with stack‐of‐stars [40], stack‐of‐spirals [41, 42], or “kooshball” [5] trajectories are available and used in pulmonary imaging research, we selected the FLORET sequence for its efficient *k*‐space coverage, which enables temporally resolved whole lung imaging while preserving sufficient parenchymal signal. Its short readout duration is particularly advantageous for imaging species with rapid T2* decay, such as lung tissue, as it minimizes signal loss and motion‐related blurring. This balance of speed, resolution, and signal preservation makes FLORET a strong choice for pulmonary imaging applications [43, 44]. In this study, the data was reconstructed at 3 mm isotropic resolution; however, this spatial resolution may limit the detection of small or subsegmental ventilation defects, particularly in patients with mild disease.

### 4.1 Limitations

A limitation of threshold analysis is the small number of healthy subjects, which did not enable balanced relations between healthy and diseased participants. Also, we have evaluated only two methods (fixed and flexible thresholds), which are both commonly used techniques in PREFUL MRI. Techniques like linear‐binning or clustering methods might yield different results.

The lack of repeatability and reproducibility data for both proton‐based techniques, derived using FLORET UTE MRI, presents a limitation of this study. In the current literature, there are several publications assessing the repeatability of signal‐based methods [32, 45], but direct comparison to volume‐based methods is missing. This could be targeted in a following study, as included subjects of this study were scanned just once and therefore no repeatability assessment was possible.

An additional limitation of this work lies in the evolving nature of registration algorithms. While the parameters used here were based on PREFUL research in numerous studies [46, 47], future improvements are expected to be made. Alternative registration software exists, such as FireANTs [48] which offers fast GPU based registrations or optical flow based methods [42, 49, 50] which may offer more accurate representations of dynamic flow.

Another limitation of this work was that we used a median filter of 7 × 7 × 7 voxels to initially remove artifacts in JVent maps. This may artificially enlarge ventilation defects, obscure smaller ones, or change their shape. Future studies should involve improving the quality of raw JVent maps using more accurate registration or increasing the spatial resolution of the MR images.

Finally, spatial matching of both proton methods with ^129^Xe MRI was not performed in this study. This step is extra challenging due to several factors, such as different breathing maneuvers (free tidal breathing vs. breath‐hold), mismatched image resolutions and field of views.

## 5 Conclusion

Both RVent and JVent methods are sufficient at detecting regional ventilation abnormalities, but RVent showed significantly stronger correlation with hyperpolarized ^129^Xe MRI. While JVent offers a simpler implementation and requires fewer additional processing steps, its reliance on accurate vector estimation during registration limits its performance. Overall, RVent appears better suited for accurate and clinically relevant assessment of lung ventilation using proton MRI.

## Funding

This work was supported by the University of Cincinnati, Office of the Vice President for Research – URC Graduate Student Stipend and Research Cost Program for Faculty—Student Collaboration (D700169), the Cincinnati Children's Research Foundation. National Organization for Rare Disorders (20003), the German Center for Lung Research (DZL), and the National Institutes of Health (2R01HL126771, R01HL143011, R01HL151588, R01HL166335).

## Conflicts of Interest

F.K., A.V., and J.V.‐C. are shareholders of BioVisioneers GmbH, a company, which has interest in pulmonary magnetic resonance imaging methods. M.M.K. is an employee of BioVisioneers GmbH, but did not receive funding for this research. M.M.W. and L.L.W. have received consulting fees from Polarean Imaging, PLC, but did not receive funding for this research project. The remaining authors have no conflicts of interest to declare.

## Supporting information


**Table S1:** VDP threshold analysis for RVent parameter. Sensitivity, specificity, Youden index, AUC, along with correlation to ^129^Xe MRI were the most important evaluated parameters.

**Table S2:** VDP threshold analysis for JVent parameter. Sensitivity, specificity, Youden index, AUC, along with correlation to ^129^Xe MRI were the most important evaluated parameters.

## Data Availability

The data that support the findings of this study are available on request from the corresponding author. The data are not publicly available due to privacy or ethical restrictions.
